# In Situ Investigation of the Formation Kinematics of Plasma-Generated Silver Nanoparticles

**DOI:** 10.3390/nano10030555

**Published:** 2020-03-19

**Authors:** Daniel Tasche, Mirco Weber, Julia Mrotzek, Christoph Gerhard, Stephan Wieneke, Wiebke Möbius, Oliver Höfft, Wolfgang Viöl

**Affiliations:** 1 Faculty of Engineering and Health, HAWK University of Applied Sciences and Arts, Von-Ossietzky-Str. 99/100, 37085 Göttingen, Germany; daniel.tasche2@hawk.de (D.T.); mirco.weber@hawk.de (M.W.); julia.mrotzek@hawk.de (J.M.); christoph.gerhard@hawk.de (C.G.); stephan.wieneke@hawk.de (S.W.); 2 Faculty of Natural and Materials Science, Clausthal University of Technology, Robert-Koch-Straße 42, 38678 Clausthal-Zellerfeld, Germany; 3 Institute of Inorganic Chemistry, Georg August University of Göttingen, Tammannstraße 4, 37077 Göttingen, Germany; 4 Department of Neurogenetics, Max Planck Institute of Experimental Medicine, Electron Microscopy Core Unit, Hermann-Rein-Str. 3, 37075 Göttingen, Germany; moebius@em.mpg.de; 5 Institute of Electrochemistry, Clausthal University of Technology, Arnold-Sommerfeld-Straße 6, 37678 Clausthal Zellerfeld, Germany; O.Hoefft@pe.tu-clausthal.de

**Keywords:** silver nanoparticles synthesis, atmospheric pressure plasma, formation kinematic

## Abstract

In this publication, it is shown how to synthesize silver nanoparticles from silver cations out of aqueous solutions by the use of an atmospheric pressure plasma source. The use of an atmospheric pressure plasma leads to a very fast reduction of silver ions in extensive solvent volumes. In order to investigate the nanoparticle synthesis process, ultraviolet/visible (UV/VIS) absorption spectra were recorded in situ. By using transmission electron microscopy and by the analysis of UV/VIS spectra, the kinetics of silver nanoparticle formation by plasma influence can be seen in more detail. For example, there are two different sections visible in the synthesis during the plasma exposure process. The first section of the synthesis is characterized by a linear formation of small spherical particles of nearly constant size. The second section is predominated by saturation effects. Here, particle faults are increasingly formed, induced by changes in the particle shape and the fusion of those particles. The plasma exposure time, therefore, determines the shape and size distribution of the nanoparticles.

## 1. Introduction

In recent years, interest in the coupling of plasmas and liquids has strongly increased for scientific and technological reasons. The synthesis of nanoparticles by means of an atmospheric pressure plasma discharge ignited above a solution has attracted attention [1]. In the areas of medicine [2], textile technology [3], printing technology [4], biotechnology [5], and sensor technology, especially surface-enhanced Raman scattering [6], the silver nanoparticle creation is of particular interest due to its versatile application. The strong antibacterial effect of silver nanoparticles is especially useful in the medical field [2].

Silver nanoparticle synthesis is usually achieved by reduction via sodium borohydride [7]. It is also possible to obtain silver nanoparticles by laser ablation of a silver solid within a solution [8], with gamma radiation, via UV-photoreduction [9], or through high-temperature reactions [10]. There is also an ecologically valuable way to synthesize silver nanoparticles of different sizes by using microalgae [11], cyanobacteria [12], plant extracts [13], enzymes [14], bacteria [15], and fungi [16]; this is the so-called green silver nanoparticle synthesis.

Microplasmas are often used for the plasma synthesis of nanoparticles under atmospheric pressure in combination with liquids [17,18] or for layer-forming processes [19]. Chemical-reducing agents can be avoided by using a plasma with the effect that the reaction times are fast [18] and the reaction can be stopped at any time by switching off the plasma power source. In addition, plasma offers a new possibility of material synthesis that is not possible with conventional methods [1].

In this publication, we investigate the synthesis of nanoscale silver particles by using a scalable plasma source. The synthesis of those particles from an aqueous silver nitrate solution is monitored in situ via ultraviolet/visible (UV/VIS) absorption spectroscopy. To investigate the kinetics of particle formation in more detail, different stock solutions and treatment times are used.

## 2. Materials and Methods

### 2.1. Formation of Silver Colloid

For the stock solution, dissolved sodium citrate (WHC GmbH, Hilgertshausen, Germany, 0.77 mg, 3 μmol, 5 Eq) in water (600 μL) was initially provided as a stabilizing agent. Afterward, 600 μL sodium citrate solution was added to an aqueous solution of silver nitrate (Carl Roth GmbH and Co. KG, Karlsruhe, Germany, ≥99.9%) for stock solution 1 (S1): 0.1 mg, 0.6 μmol, 1 Eq; for stock solution 2 (S2): 0.3 mg, 1.8 μmol, 3 Eq; for stock solution 3 (S3): 0.5 mg, 3 μmol, 5 Eq; for stock solution 4 (S4): 0.7 mg, 4.2 μmol, 7 Eq. The stock solutions were generated shortly before plasma treatment, and they are stable and transparent for three months without plasma treatment. 

### 2.2. Plasma Treatment

**Figure 1:**
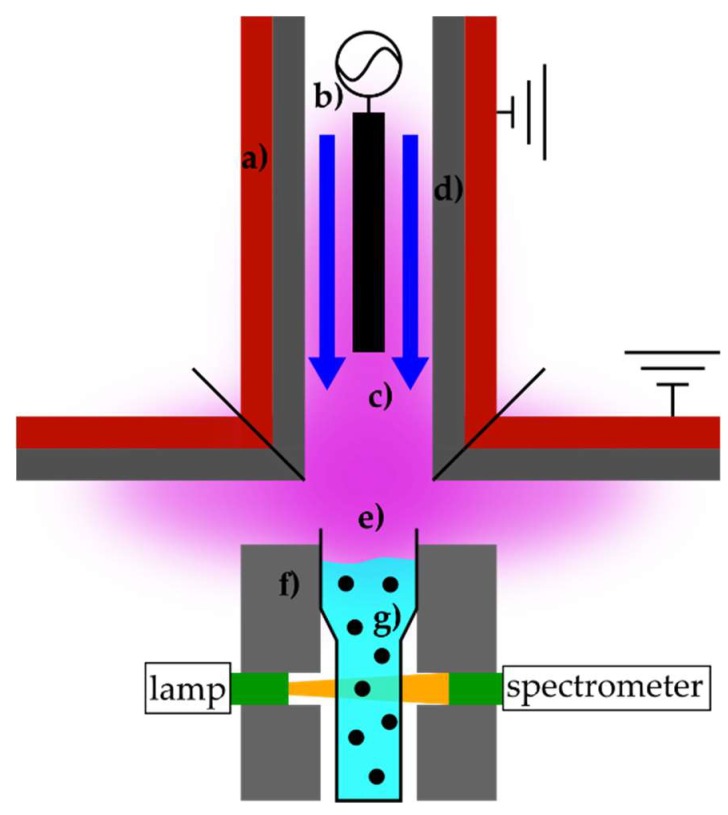
Schematic illustration of silver nanoparticle synthesis using plasma source consisting of (**a**) ground electrode, (**b**) high-voltage electrode, (**c**) working gas flow, (**d**) dielectric material, (**e**) plasma discharge, (**f**) cuvette mount with connections to the spectrometer, and (**g**) UV cuvette with stock solutions.

**Figure 2:**
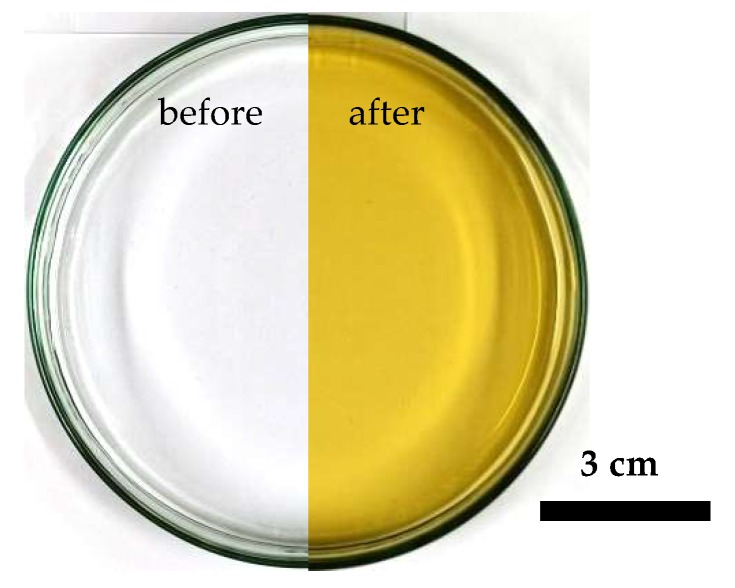
Representation of upscaling feasibility tests with the used plasma source. The untreated stock solution S2 (**left**) is compared to stock solutions S2 after a plasma treatment time of 60 s (**right**) in this illustration. In this upscaling experiment, the treatment area was 250 cm² and the volume was 30 mL.

The setup to generate and analyze the nanoparticles is illustrated in Figure 1. The used plasma source, which is described in more detail in [20], generates a non-thermal atmospheric pressure plasma based on a dielectric barrier discharge. The high-voltage electrode is made of stainless steel and the ground electrode of copper, which is coated with aluminum oxide (Al_2_O_3_). In this setup, the water surface is an active part of the plasma discharge, forming a surface barrier discharge on the liquid phase. The working distance between the solution surface and the plasma source was fixed at 11 mm. As a working gas, argon 5.0 (Linde AG, Pullach, Germany, ≥99.999%) with a flow rate of 30 L/min and, as a high-voltage source, a plasma generator HV-X20 (Tantec, Lunderskov, Denmark) were used. This generator operated with a measured input power of *P* ≈ 160 W, a frequency of *f* ≈ 14.5 kHz, and a power density of *P_density_* ≈ 4 W/cm^3^ in a plasma volume of about 40 cm^3^ above and around the polymer material UV-cuvette (Brand GmbH and Co. KG, Wertheim, Germany, 230–900 nm, *d_cuvette_* = 1 cm). The plasma source was cylindrical with a diameter of 80 mm, which can be used above a large area for the synthesis of nanoparticles. To illustrate the scalability of the plasma treatment, Figure 2 shows a 60 s plasma-treated stock solution S2 with a volume of 30 mL and a surface area of about 250 cm². The homogeneous effect of the plasma treatment can be recognized. The coloring of the stock solution resulting from the plasma treatment is explained in Section 3.

### 2.3. Plasma Diagnostics

In order to characterize the plasma discharge regarding rotational temperature, vibrational temperature, and electron density, optical emission spectroscopy (OES) was used. These parameters offer an insight into the collisional parameters of the plasma and offer the possibility of classifying the used plasma source. The spectra were obtained using an Échelle-Spectrometer Aryelle-Butterfly 400 (LTB Lasertechnik Berlin GmbH, Berlin, Germany), calibrated to wavelength and relative intensity, with a wavelength resolution of <80 pm. The optical fiber was placed perpendicular to the plasma effluent so that the plasma emission directly above the surface of the analyzed solution was investigated. For further diagnostics, five spectra between 300 and 960 nm, each with an integration time of 100 ms, were taken at room temperature and ambient air (20 °C; 36% relative humidity), dark-corrected, and integrated.

The resulting spectrum shows reactive ArI species, OH (A-X), N_2_ (*C*^3^Π_u_ − *B*^3^Π_g_), H_α_, and OI, whereas higher excited species are absent. Therefore, OH, N_2_, H_α_, and OI most probably originate from the surrounding air and—to a lower extent—to impurities from the working gas.

To determine the rotational temperature, the Boltzmann-plot method, using Q1 branch lines of the OH (A-X) ro-vibrational band, was employed. Necessary parameters were taken from Chidsey et al. [21]. Additionally, the N_2_-rotational temperature, as well as N_2_-vibrational temperature, was derived, using the methods described by Peters et al. [22]. The Stark broadening mechanism of H_α_ was used in order to determine the electron density [23]. The evaluated plasma parameters are summarized in Table 1.

### 2.4. Investigation of Formation Kinetics

To determine the kinetics of nanoparticle synthesis using an atmospheric pressure plasma, UV/VIS absorption spectra were recorded in situ during the plasma treatment. For those measurements, the UV/VIS spectrometer AvaSpec-UL3648 (Avantes, Apeldoorn, The Netherlands) with a deuterium halogen lamp AvaLight-DH-S (Avantes, Apeldoorn, The Netherlands) was used. The spectra were recorded every 0.5 s with an integration time of 1.5 ms and averaged over 50 spectra using the software AvaSoft 8.9 (Avantes, Apeldoorn, The Netherlands). The plasma treatment and, thus, the nanoparticle synthesis took place in a UV cuvette for durations of 5, 10, 20, 30, 60, and 120 s. For the analysis, the spectra were smoothed by a Savitsky–Golay filter and, afterward, evaluated using peak analysis via Origin 2018 (OriginLab Corporation, Northampton, MA, USA).

In order to obtain nanoparticle samples suitable for transmission electron microscopy (TEM), grids (100 mesh hexagonal copper covered with a formvar film) were incubated with droplets of different nanoparticle solutions. Those droplets were dried by carefully absorbing the excess liquid with a piece of filter paper. Then, the nanoparticles were imaged with a LEO912 transmission electron microscope (Carl Zeiss Microscopy, Oberkochen, Germany) by using an on-axis 2k CCD camera (TRS, Moorenweis, Germany). The particle sizes were measured automatically with the software ImageJ 1.52p [24].

Furthermore, the pH-value of the solutions was measured using pH-meter FiveGo F2 with an InLab Flex-Micro electrode (Mettler Toledo, Columbus, OH, USA). In addition, the sample temperature was determined immediately after switching off the plasma with the thermal imaging camera TiS (Fluke Corporation, Everett, WA, USA).

## 3. Results and Discussion

**Figure 3:**
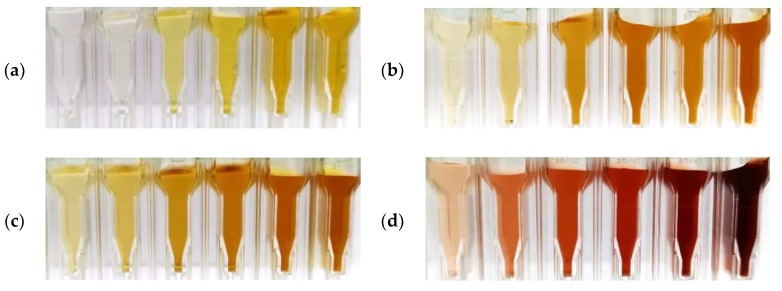
Coloration comparison of the stock solutions (**a**) S1, (**b**) S2, (**c**) S3, and (**d**) S4 after plasma treatment in UV cuvettes over an ascending plasma treatment time *t* rising from left to right (5, 10, 20, 30, 60, 120 s).

The coloration of the stock solutions after different plasma treatment times is shown in Figure 3. A yellow tint is typical for silver nanoparticles [25] and changes over the plasma treatment time and concentration of the stock solution. For the highly concentrated S4, the coloration shifts to reddish. The optical properties of silver nanoparticles in solution are dependent on particle size [26,27,28], particle material [25,29], particle shape [30,31], particle coating [32,33], particle stabilization [34], and particle environment [30,35].

### 3.1. Synthesis Pathways

In order to gain more detailed insights into the formation of silver nanoparticles, some process-determining kinetic parameters and the synthesis routes are explained below. The coloration of the stock solution by the plasma is an indication of the formation of silver nanoparticles [25]. The generated electrons, ions, radicals, and electromagnetic fields provided at the plasma/solution interface lead to the reduction of metal ions dissolved in water and ultimately to the nucleation and formation of nanoparticles [6,18]. In the literature, the formation of silver nanoparticles via plasma out of a salt solution is mainly attributed directly to emitted electrons from the plasma discharge [18] and solvated electrons [36,37]:

(1) $${Ag}^{+}+e^{-}\to {Ag}^{0}$$

By contrast, nanoscale gold particles are primarily formed by plasma ion radiation [38] or plasma-induced liquid chemistry [39]. From the investigations presented in this publication, the influence of long-living species and liquid chemistry on the reduction of silver ions is low or not process-determining. This assumption is supported by the fact that there is no significant change in absorbance in the near future after switching off the plasma.

During the whole treatment time, the pH-value stays constant (6.2 ± 0.1). Kondeti et al. [40] showed that a constant pH-value is a strong indicator for a direct reduction without reacting mainly via intermediates like H^−^, H^●^, or H_2_O_2_. A citrate-based stabilization system was used, which can also create buffer systems, that keep the pH-value in a constant range. These two circumstances made it almost impossible to estimate whether intermediate species formed or not. There is still less knowledge about the concrete reduction pathways. Some research groups claim that the reduction is realized by solvated electrons. In fact, this means that the electrons show strong interactions with the lowest unoccupied molecular orbital (LUMO) of the solvent molecules in the water [36]. Therefore, this reaction pathway is probably responsive for more or less direct reactions between the plasma discharge and the silver ions, whereby the solvent molecules serve as an electron carrier.

Other influences on particle synthesis by (i) UV radiation, (ii) temperature, and (iii) the stabilizer sodium citrate as a reducing agent are discussed beloi) A 3 mm-thick fused silica (SiO_2_) and also a 5 mm-thick window of calcium fluoride (CaF_2_) were positioned above the stock solution. The two glasses differ in their transmission characteristics, which allows the effect of UV radiation on the solution to be observed in defined wavelength ranges. In this modification of the experimental setup, the electrons, ions, and radicals generated in the plasma were excluded and only the UV radiation could interact with the stock solution. While using the SiO_2_-window, the stock solution remained unchanged. The use of the CaF_2_-window showed that the UV radiation in the wavelength range of 130–190 nm emitted from the plasma source is capable of reducing silver ions. When the stock solution was brought into direct contact with the CaF_2_-window, a silver mirror formed after a plasma treatment time of *t* = 120 s. If there is a volume of air between the window and the solution, a thin layer of a deep yellow veil formed after *t* = 120 s, which was not comparable to the result generated by the direct plasma treatment. This circumstance can be explained partly due to the absence of convection mechanisms [40]. It cannot be completely excluded that electrons were formed by the plasma discharge in the air gap between the window and the solution, which could lead to a reduction process of the silver ions. The low formation rate of silver nanoparticles is potentially an indicator for generating a small number of free electrons in the air gap.

(ii) The used plasma source generates a cold, atmospheric non-equilibrium plasma. After a treatment time of *t* = 120 s, the treated liquid surface heated up by about 5 K. This temperature increase does not explain the fast and controlled silver nanoparticle synthesis.

(iii) The stabilizer sodium citrate is not capable of acting as a reducing agent under the experimental conditions. Therefore, the plasma activation of the pure stabilizer solution and subsequent addition to the silver nitrate solution also did not lead to the formation of silver nanoparticles.

### 3.2. Form and Position of the Plasmon Resonance Peak

**Figure 4:**
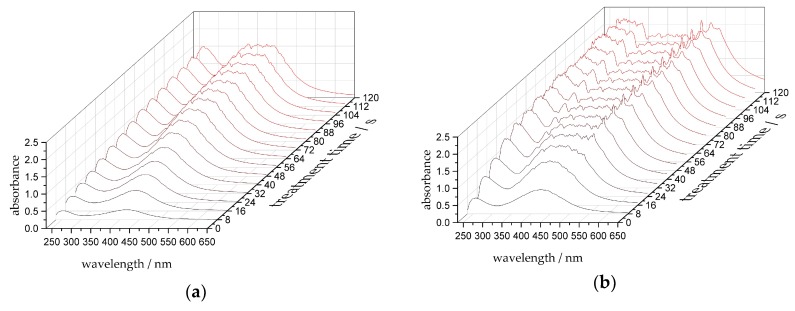
Absorbance spectra over treatment time *t*, displayed every 8 s up to a maximum treatment time of *t* = 120 s for (**a**) S1 and (**b**) S4.

In Figure 4, the progression of the spectral absorbance of the samples S1 and S4 up to a plasma treatment time *t* = 120 s is shown. The typical silver nanoparticle plasmon resonance peak above 400 nm increases with the treatment time *t*. The position of the plasmon resonance peak serves as a benchmark of the nanoparticle size [41]. For the stock solution S1, the maximum wavelength of the plasmon resonance peak is roughly at 412 nm. In S4, the resonance peak is approximately at 423 nm and, thus, larger nanoparticles were created [41].

A further peak appears around 255 nm, which is a signal of the solution [10]. This peak also increases with the plasma treatment time *t*, which is due to the change in the chemical composition inside the solution. In this wavelength range, nitrites NO_2_^−^, nitrates NO_3_^−^ [42,43], and hydrogen peroxide H_2_O_2_ [44] can contribute to the signal, which indicates their formation in the stock solution during the plasma treatment.

As the plasma treatment time *t* increases, the plasmon resonance signal becomes broader. A suitable explanation could be a broadening caused by a larger particle size distribution [27], aggregation processes of the created nanoparticles [45], or changes in particle shape [30]. The signal broadening depends on the composition of the stock solution and the ratio between silver and citrate ions, respectively. The stock solution S4 shifts to the reddish wavelength range, mainly attributed to agglomeration processes between the nanoparticles, caused by insufficient stabilization due to the high ratio of silver ions to citrate ions of 7:5 [45,46].

**Figure 5:**
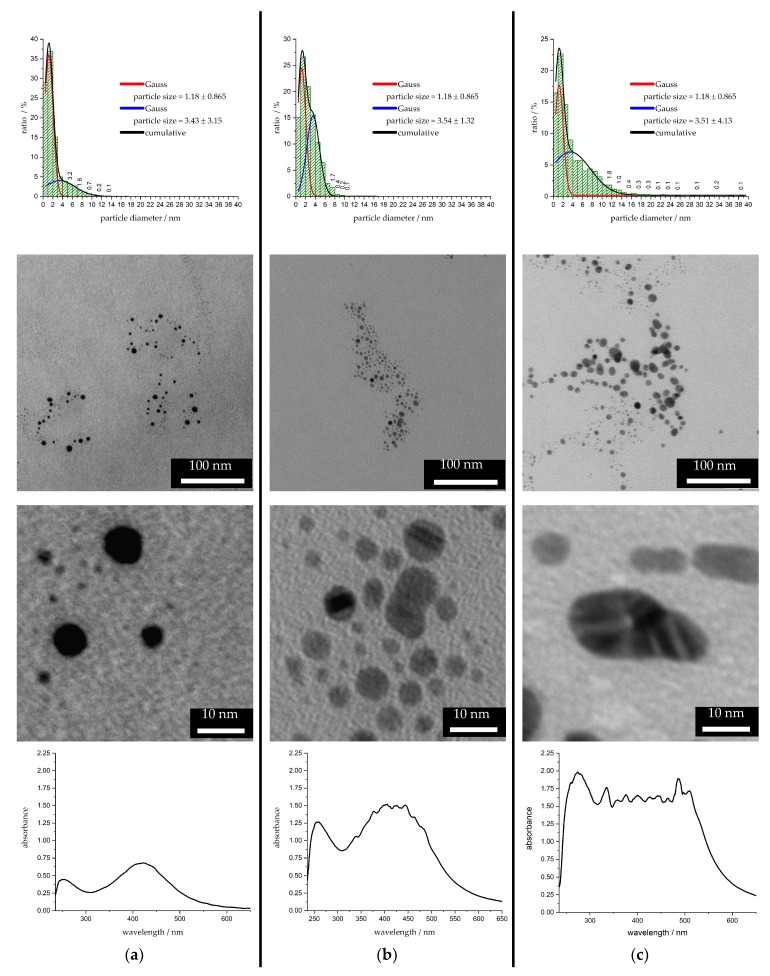
Particle size distribution fitted by Gaussian functions with mean particle size and standard deviation illustrated at the top. UV/VIS absorption spectra and characteristics of the particles are shown at the bottom with TEM-images in between. On the left-hand side: (**a**) S2 after plasma treatment time of *t* = 10 s, in the middle: (**b**) S2 after *t* = 30 s, and on the right-hand side: (**c**) S4 after *t* = 60 s are shown.

Imaging via transmission electron microscopy (TEM) was used to check the possible aggregation, particle size distribution, and particle shape. TEM images of different stock solutions containing nanoparticles after different treatment times *t* with corresponding particle size histograms and UV/VIS absorption spectra are illustrated in Figure 5. It is recognizable that at a treatment time of *t* = 10 s, the particles in stock solution S2 are spherical and the majority of the particles are in the size range of *d_particle_* ≤ 2 nm. In addition, some spherical nanoparticles with particle diameters of *d_particle_* ≤ 10 nm, which possess a higher optical activity [47], are already present. The characteristics of the UV/VIS absorption signal after *t* = 10 s are narrow and show a defined shape; these characteristics also symbolize the observed attributes of the formed nanoparticles.

With longer treatment times *t*, the silver nuclei and small nanoparticles grew to larger silver nanoparticles [48,49] in the stock solution S2. At a treatment time of *t* = 30 s, particle–particle unions, staple faults [50], twinned crystals [48], and also non-spherical nanoparticles appeared. Crystallization processes leading to the resulting crystal structure of silver [48] cause the change in particle shape. After a treatment time of *t* = 60 s for stock solution S4, particle–particle unions, non-spherical nanoparticles, and staple faults occur more frequently. Particles with a maximum particle diameter of *d_particle_* ≈ 130 nm are formed. For large silver nanoparticles, an extinction wavelength around 600 nm [45] results, which explains the redshift of the spectrum. For the two longer treated stock solutions, the UV/VIS absorption spectra clearly show the diversity of the resulting particles in shape and size.

By fitting the histogram data with two Gauss functions, conclusions are drawn about the synthesis steps nucleation and particle growth. The two Gauss functions symbolize different size regimes. The first Gauss function describes very small nanoparticles or nuclei with a particle diameter *d*_particle_ in the range around 2 nm. The position and width of the first Gauss function are determined after a plasma treatment time *t* = 10 s and are then kept constant for further analysis. The second Gauss function describes larger particles and agglomerates. The start of nucleation and growth of small nanoparticles is included in the first size regime and, therefore, in the first Gauss function. With the treatment time *t*, the small size regime decreases and the nanoparticles grow. Over the treatment time from *t* = 10 to 60 s, the area below the first Gauss function decreased approximately to the half value and the area below the second Gauss function increased to the double value. This means that the occupation of the tiny particle state disappears constantly and the occupation of the state of larger particles and agglomerates grows at the same rate [49]. The small size regime does not empty itself completely as new nuclei continue to be formed by plasma discharge. The plasma also provides strong alternating electric fields and fast electrons, which may lead to the disintegration of large nanoparticles, nuclei, or agglomerates [51]. In addition, it is conceivable that larger particles grow preferentially, whereby smaller particles grow less in size. Unfortunately, no conclusions can be drawn from the experimental data in this context.

### 3.3. Absorbance

**Figure 6:**
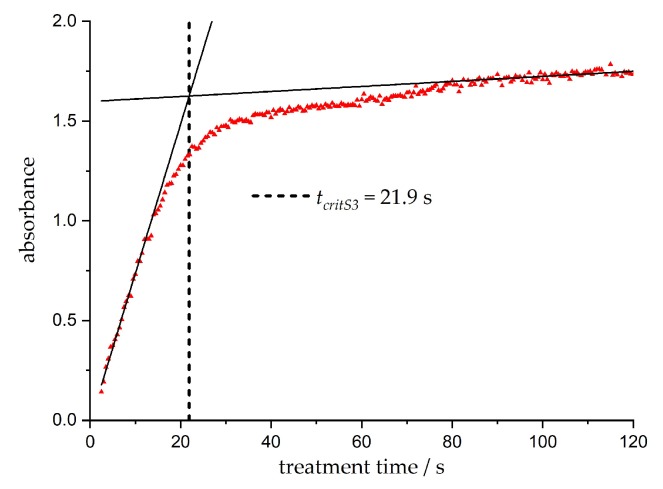
Absorbance over a treatment time of *t* = 120 s for stock solution S3 and resulting determination of the critical time *t_crit_* for plasma treatment.

The absorbance of silver plasmon resonance peaks increases with the plasma treatment time *t*, as shown in Figure 6 for stock solution S2. The absorbance *A* is described by the Lambert–Beer law:

(2) $$A=c\cdot L\cdot \varepsilon \left(d_{particle}\right),$$

and depends on the measuring distance *L*, the concentration of nanoparticles *c*, and the absorbance coefficient *ε(d_particle_)*. The absorbance coefficient *ε(d_particle_)* for silver nanoparticles is strongly and not linearly dependent on the particle size *d_particle_* [47]. Over the treatment time *t,* the concentration *c* of the nanoparticles and the absorbance coefficients *ε(d_particle_)* change due to particle growth and nucleation. The absorbance *A* is measured within the wavelength range of 415–425 nm depending on the stock solution.

First, the absorbance increases linearly with the treatment time *t* and then shifts into saturation area. The critical treatment time *t_crit_* results from the intersection of two linear regressions; on the one hand, from the first 10 s of treatment (linear growth), and on the other hand, from the last 20 treatment seconds (linear saturation). For the sample S3, this is shown as an example in Figure 6. Up to *t_crit_*, a linear synthesis behavior is assumed within good approximation. The critical treatment times *t_crit_* for the different stock solutions are summarized in Table 2. In combination with the explanations made in the previous section, the conclusion is drawn that mainly spherical small particles were formed up to *d_particle_* ≤ 10 nm by the plasma before *t_crit_* was reached. After *t_crit_* has passed, non-linear effects of particle growth such as agglomeration, particle staple faults, or particle shape changes dominate, which broaden the UV/VIS absorption signal rather than change the absorbance. Therefore, the colloids and synthesis become more indefinite and harder to describe in detail. Similar observations and conclusions regarding the phases of nucleation and growth via changes in the UV/VIS absorption spectrum like saturation and linear growth were made by Polte et al. [49] during the chemical synthesis of silver nanoparticles.

### 3.4. Reaction Rate Coefficient

A parameter of the reaction kinetics is the reaction rate coefficient *k* of the silver nanoparticle synthesis, which can be determined by in situ UV/VIS spectrometry. For this purpose, the following simplifications have to be applied. The reduction of silver ions is mainly affected directly by electrons, according to Equation (1). The plasma provides a large amount of electrons for the reduction of silver ions at the interface between liquid and plasma, which is the reason why the reaction corresponds to a pseudo-first order:

(3) $${\mathrm{Ag}}^{+}\;\to {\;\mathrm{Ag}}^{0}.$$

This allows the reaction to be determined by

(4) $$c_{t}\left({\mathrm{Ag}}^{+}\right)=c_{0}\left({\mathrm{Ag}}^{+}\right)\cdot e^{-\mathrm{kt}},$$

where *k* is the reaction rate coefficient, *c_t_*(Ag^+^) is the silver ion concentration at a treatment time *t*, and *c_0_*(Ag^+^) is the initial silver ion concentration before treatment. The reaction rate coefficient *k* can be obtained by [52]

(5) $$k=-\ln\left(\frac{c_{t}\left({\mathrm{Ag}}^{+}\right)}{c_{0}\left({\mathrm{Ag}}^{+}\right)}\right)\cdot \frac{1}{t},$$

(6) $$k=-\ln\left(\frac{c_{max}\left({\mathrm{Ag}}^{0}\right)-c_{t}\left({\mathrm{Ag}}^{0}\right)}{c_{max}\left({\mathrm{Ag}}^{0}\right)}\right)\cdot \frac{1}{t}.$$

Assuming that the silver ion concentration is dependent on and, hence, approximately proportional to the absorbance *A* of the Lambert–Beer law (Equation (2)), *k* results in

(7) $$k=-\ln\left(\frac{A_{max}-A_{t}}{A_{max}}\right)\cdot \frac{1}{t},$$

with *A_max_* for the maximum absorbance and *A_t_* as absorbance at a treatment time *t*. However, it should be mentioned that the applied model does not consider all contributing phenomena such as diffusion of silver ions from the liquid volume to its surface. It is, thus, an idealized approach for the description of the process.

The slope of the linear regression taken from the Arrhenius plot in Equation (6) over the plasma treatment time *t* to *t_crit_* results in the reaction rate coefficient *k*, summarized for the various stock solutions in Table 1. The reaction rate *k* in the linear synthesis range increases more strongly if the silver nitrate concentration of the stock solution is very high and vice versa.

## 4. Conclusions

The reduction of silver ions and the generation of nanoparticles were realized successfully by means of an atmospheric pressure plasma source. It was possible to observe the formation kinetics of silver nanoparticles in situ via UV/VIS-spectroscopy in a detailed temporal resolution. By transmission electron microscopy (TEM), the time-dependent alterations of the nanoparticle shape could be illustrated properly. The shape and number distribution were found to be in good accordance with the absorbance signals from the UV/VIS spectra. With this knowledge, clues arose to estimate the particle formation process in two different main synthesis phases within the considered time range. The analytical method of in situ UV/VIS-spectroscopy can be used to control the atmospheric pressure plasma reduction process in order to gain silver nanoparticles in a specifically defined quality.

For future research, the influence of plasma parameters such as working gas, power input, and working distance on nanoparticle synthesis should be evaluated by in situ UV/VIS spectrometry. In a further step, the plasma-generated nanoparticles could be applied in situ on fiber materials such as non-woven fabrics, especially to make use of the antimicrobial properties of silver nanoparticles in this case. Due to its scalability, the plasma source usage is excellently suitable for possible industrial applications.

## Figures and Tables

**Table 1:** Temperatures and electron density inside the plasma discharge.

Parameter	Value
OH-rotational temperature	669 ± 134 K
N_2_-rotational temperature	820 ± 50 K
N_2_-vibrational temperature	3017 ± 742 K
Electron density	3.8 × 10^21^ ± 2.4 × 10^21^ m^−3^

**Table 2:** Critical treatment times *t_crit_* and reaction rate coefficients *k* for the different stock solutions.

Parameter	S1	S2	S3	S4
*t_crit_* in s	36.8	23.4	21.9	18.7
*k* in s^−1^	0.11	0.15	0.19	0.22
